# Modelling and Validation of Computer Vision Techniques to Assess Heart Rate, Eye Temperature, Ear-Base Temperature and Respiration Rate in Cattle

**DOI:** 10.3390/ani9121089

**Published:** 2019-12-06

**Authors:** Maria Jorquera-Chavez, Sigfredo Fuentes, Frank R. Dunshea, Robyn D. Warner, Tomas Poblete, Ellen C. Jongman

**Affiliations:** 1 Faculty of Veterinary and Agricultural Sciences, University of Melbourne, Melbourne, VIC 3010, Australia; sigfredo.fuentes@unimelb.edu.au (S.F.); fdunshea@unimelb.edu.au (F.R.D.); robyn.warner@unimelb.edu.au (R.D.W.); totopoblete@gmail.com (T.P.); 2 Animal Welfare Science Centre, Faculty of Veterinary and Agricultural Sciences, University of Melbourne, Parkville, VIC 3010, Australia; ejongman@unimelb.edu.au

**Keywords:** computer vision, physiological parameters, animal monitoring, imagery

## Abstract

**Simple Summary:**

Animal monitoring normally requires procedures that are time- and labour-consuming. The implementation of novel non-invasive technologies could be a good approach to monitor animal health and welfare. This study aimed to evaluate the use of images and computer-based methods to track specific features of the face and to assess temperature; respiration rate and heart rate in cattle. The measurements were compared with measures obtained with conventional methods during the same time period. The data were collected from ten dairy cows that were recorded during six handling procedures across two consecutive days. The results from this study show over 92% of accuracy from the computer algorithm that was developed to track the areas selected on the videos collected. In addition, acceptable correlation was observed between the temperature calculated from thermal infrared images and temperature collected using intravaginal loggers. Moreover, there was acceptable correlation between the respiration rate calculated from infrared videos and from visual observation. Furthermore, a low to high relationship was found between the heart rate obtained from videos and from attached monitors. The study also showed that both the position of the cameras and the area analysed on the images are very important, as both had large impact on the accuracy of the methods. The positive outcomes and the limitations observed in this study suggest the need for further research

**Abstract:**

Precision livestock farming has emerged with the aim of providing detailed information to detect and reduce problems related to animal management. This study aimed to develop and validate computer vision techniques to track required features of cattle face and to remotely assess eye temperature, ear-base temperature, respiration rate, and heart rate in cattle. Ten dairy cows were recorded during six handling procedures across two consecutive days using thermal infrared cameras and RGB (red, green, blue) video cameras. Simultaneously, core body temperature, respiration rate and heart rate were measured using more conventional ‘invasive’ methods to be compared with the data obtained with the proposed algorithms. The feature tracking algorithm, developed to improve image processing, showed an accuracy between 92% and 95% when tracking different areas of the face of cows. The results of this study also show correlation coefficients up to 0.99 between temperature measures obtained invasively and those obtained remotely, with the highest values achieved when the analysis was performed within individual cows. In the case of respiration rate, a positive correlation (r = 0.87) was found between visual observations and the analysis of non-radiometric infrared videos. Low to high correlation coefficients were found between the heart rates (0.09–0.99) obtained from attached monitors and from the proposed method. Furthermore, camera location and the area analysed appear to have a relevant impact on the performance of the proposed techniques. This study shows positive outcomes from the proposed computer vision techniques when measuring physiological parameters. Further research is needed to automate and improve these techniques to measure physiological changes in farm animals considering their individual characteristics.

## 1. Introduction

The livestock industry is continually seeking more sustainable systems, which implement novel technologies that contribute to better management strategies [1]. Precision livestock farming (PLF) has emerged as a response to the need for a regular monitoring system that provides detailed information, allowing quick and evidence-based decisions on the animal’s needs [2]. Sound sensors, cameras, and image analysis have been considered as part of the PLF development, which appear to be useful non-invasive technologies that allow the monitoring of animals without producing discomfort [3,4,5,6,7]. 

Some researchers are also developing and implementing new technologies in order to achieve automatic and less invasive techniques to monitor vital parameters such as heart rate (HR), respiration rate (RR) and core body temperature [8,9,10,11]. These parameters have been commonly measured through methods that require human–animal interaction, such as the use of stethoscope for HR and RR measurement, and thermometer for core body temperature measurement [12,13,14]. Although these and other contact techniques have been widely used for medical, routine and scientific monitoring of animals, most of them are labour-intensive, can generate discomfort to animals and consequently, they are not practical for continuous and large-scale animal monitoring [15,16]. Hence, computer vision (CV) techniques appear as promising methods to perform non-contact measuring of one or more physiological parameters, including temperature, HR, and RR in farm animals [17]. 

Thermal infrared (TIR) sensors have been used for decades in research aiming to assess skin temperature as an indicator of health and welfare issues in farm animals [11]. The implementation of these technologies is based on the fact that the alteration of blood flow underlying the skin generates changes in body surface temperature, which is detected as radiated energy (photons) by TIR cameras, and later observed as skin temperature when analysing the images [18]. This analysis allows the selection of one or more regions of interest (ROIs), which has led to the use of these technologies to detect signs of inflammation in certain areas of the body, or to assess the surface temperature of specific areas, as an index to body temperature in animals [19,20]. 

In terms of HR measurement, a variety of less invasive techniques, such as attached monitors, have been tested to assess HR in cattle and other farm animals. However, it has been found that this measurement can be affected by the equipment and the measurement technique [21]. For this reason, researchers are currently investigating computer-based techniques as a promising possibility for a contactless way of monitoring HR on farms [22]. This interest is also promoted by the results reported in humans, which have shown the ability to detect changes in blood flow, which allows the measurement of HR in people by using images and computer algorithms that are based on skin colour changes or the subtle motion that occurs in the body due to cardiac movements [23,24,25,26,27]. Although these studies showed promising results, most of them agree that the main noise observed in the results are related to motion and light conditions [25]. Some researchers such as Li et al. [28] and Wang et al. [29], have focused on improving these methods by including algorithms for tracking the ROI and pixels, and for illumination correction. 

In terms of RR, as another important measurement used in human and animal health, the search for new and less labour-intensive methods of assessment has grown in the last decades. Methods such as respiratory belt transducers, ECG (electrocardiogram) morphology, and photoplethysmography morphology have been implemented in several studies [30]. However, these methods require the attachment of sensors, which can produce discomfort and stress in the person or animal [30,31]. Hence, the use of imagery and computer algorithms has increasingly been used in studies aiming to remotely assess the RR of people and animals [5,17,30,31]. Although these studies showed promising results when remotely measuring RR, they were only tested when the participants were motionless, or required to count the respiration movements manually. 

This study aimed to develop and validate computer vision algorithms against more traditional measurements to measure (1) ‘eye’ and ‘ear-base’ temperature from TIR imagery, (2) RR from infrared videos, (3) HR from RGB videos, and (4) an algorithm for feature tracking in cattle. Additional aims were to identify the most suitable camera position and the most suitable ROI, both in terms of the validity as well as accuracy of the proposed algorithms, to facilitate possible implementation of these methods in an automated monitoring system on farm. 

## 2. Materials and Methods 

### 2.1. Study Site and Animals Description

This study was approved by The University of Melbourne’s Animal Ethics Committee (Ethics ID: 1714124.1). Data was collected during two consecutive days at the University of Melbourne’s robotic dairy farm (Dookie, VIC, Australia) in March 2017. Ten lactating cows (Holstein Friesian) varying in parity (2–5), average of daily milk production (32.2–42.3 kg), and liveweight (601–783 kg), were randomly selected from the herd. These cows were placed in a holding yard the night before starting the procedures and kept in this yard overnight during the experiment period.

### 2.2. Data Acquisition and Computer Vision Analysis

#### 2.2.1. Cameras Description

Devices equipped with thermal and RGB sensors were built, allowing to simultaneously record thermal and RGB images of the animals involved. The sensors incorporated were an infrared thermal camera (FLIR^®^ AX8; FLIR Systems, Wilsonville, OR. USA) and an RGB video camera (Raspberry Pi Camera Module V2.1; Raspberry Pi Foundation, Cambridge, UK).

The FLIR AX8 Thermal Camera has a spectra range of 7.5–13 μm and accuracy of ±2% The emissivity used in these cameras is 0.985, which has been reported as the emissivity of humans and other mammals skin (0.98 ± 0.01; [6,32]). Thermal imaging cameras with similar characteristics have been used in several studies to assess skin temperature in humans and animals [6,33,34,35,36]. Furthermore, the RGB video camera used for this project was a Raspberry Pi Camera Module V2.1, which is an 8-megapixel sensor, with a rate of 29 frames per second.

In addition to the built devices, two Hero 3+ cameras (GoPro, Silver Edition v03.02^®^, San Mateo, CA, USA) were attached to the milking robot to continuously record the side and front area of the cows face while they were being milked. These cameras record RGB videos with a rate of 29 frames per second, with a resolution of 1280 × 720 pixels. Moreover, a FLIR^®^ ONE (FLIR Systems, Wilsonville, OR, USA) Thermal Imaging Case attached to an iPhone^®^ 5 (Apple Inc., Cupertino, CA, USA) was used to record non-radiometric IR videos when animals were in the crush.

#### 2.2.2. Feature Tracking Technique

This methodology was developed to avoid the misclassification of pixels that could later be considered for the physiological parameters prediction. The feature tracking procedure aimed to trace through sequential frames of a video, characteristic points over an ROI using computer vision algorithms. The complete feature tracking methodology was integrated on a graphical user interface built in Matlab^®^ R2018b, which is divided into two main steps: (i) identification of features over the ROI and ii) reconstruction of a new ROI based on the tracked point over frames. 

**Figure 1:**
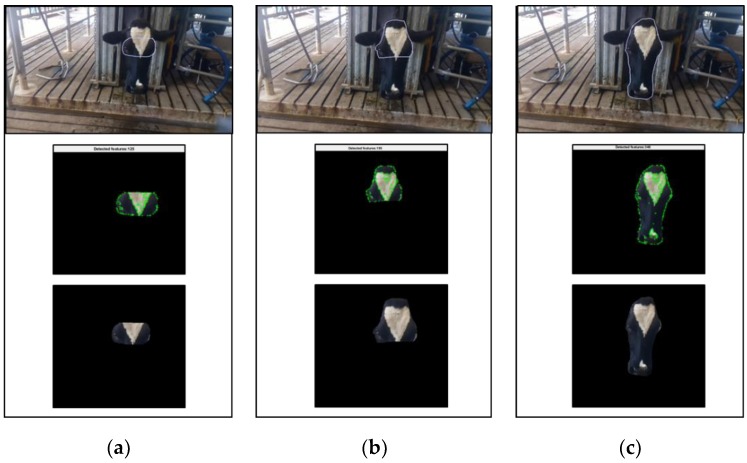
Stages of the feature tracking method when cameras are located in front of the animal on (**a**) the eye area, (**b**) the forehead and (**c**) the face.

**Figure 2:**
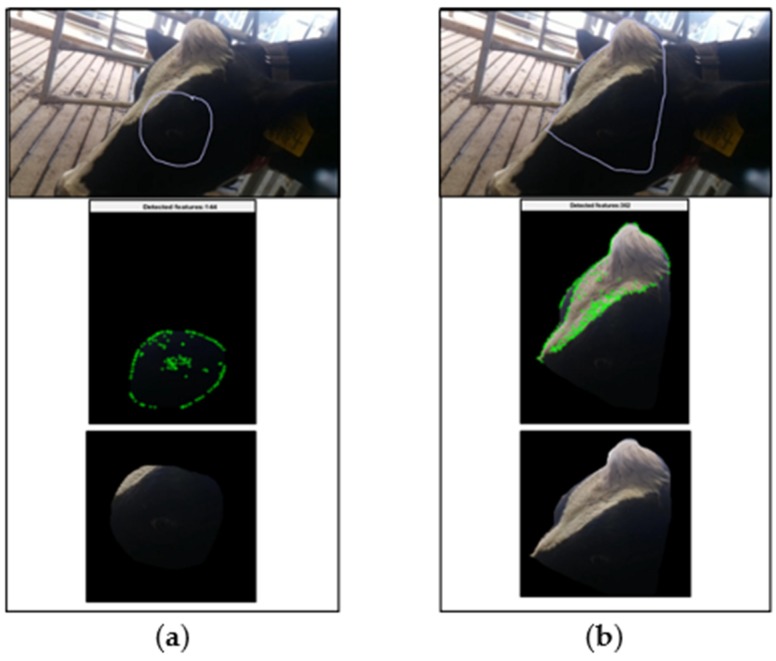
Stages of the feature tracking method when cameras are located next to the animal on (**a**) the eye area and (**b**) the forehead.

(i) For the feature identification step, the following pattern recognition techniques are automatically computed: the minimum eigenvalue [37], the speeded up robust features (SURF) [38], the binary robust invariant scalable points (BRISK) [39], the fusing points and lines for high performance tracking (FAST) algorithm [40], a combined corner and edge detection algorithm [41], and the histograms of oriented gradients algorithm [42]. As these techniques differ in performance and the target for feature detection, they are automatically compared within the ROI of each specific video. After these techniques have been evaluated, the one that allows to select the most representative features, for each case, is selected. Some of the functions developed as part of the methodology were based on the Computer Vision Toolbox available on Matlab^®^ R2018b.

(ii) With the results from the previous step, the selected feature identification technique and the selected ROI are used for tracking the points over the sequential frames, using a modified KLT (Kanade–Lucas–Tomasi)-based algorithm [43]. This tracking procedure refreshes the selected ROI over all the consecutive frames and set up a new ROI based on the area which better includes the new identified points by masking (using a binary image segmentation) all the pixels which are outside of that region (Figure 1 and Figure 2). This new ROI is only created if the ratio between the selected point and the new identified points is higher than 70%. Finally, the physiological parameters prediction was applied over the frames containing only the information of interest.

This method decreased the misclassification of pixels by increasing accuracy when analyzing the desired area. Another advantage of this methodology is that as it is automated (not supervised), it only requires the user input for the initial selection of the ROI and for the selection of the feature identification technique that better represents the features of the ROI.

#### 2.2.3. Image Analysis

Image processing was performed separately for each physiological parameter. In the case of temperature, TIR images, obtained with FLIR AX8 cameras, were processed to extract temperature. To automatize the processing of the thermal images, a script was developed on MATLAB^®^ R2018b (Mathworks Inc. Natick, MA, USA), using a software development kit (SDK) named FLIR^®^ Atlas SDK [44,45]. The script consisted on automatically extracting the radiometric information from the original data and saving it into ‘.tiff and .dat’ files. In addition, a ROI was set on the images for extracting the statistical parameters of the temperature on the specific area selected. In this study ‘eye area’ and ‘ear base’ were used as ROI to extract temperature. The selection of these two areas was based on some reported studies, which analysed these areas when assessing temperature of farm animals as a measurement of body temperature [46,47].

The non-radiometric infrared (IR) videos obtained with the FLIR ONE were processed through an algorithm that was developed in Matlab^®^ for this study, to measure the RR in the participating cows. This algorithm calculates RR measures based on the changes in pixel intensity values detected within the ROI (nose area) due to respiratory airflow, which indicates the inhalations and exhalations performed by the animal during the time analysed. 

For the HR analysis, RGB videos obtained with Raspberry Pi and GoPro cameras were firstly examined and classified per cow. Once the videos of each animal were identified, they were processed through a customized algorithm developed in Matlab^®^ 2018b, which identifies the changes of luminosity in the green colour channel from ROIs obtained, using the photoplethysmography principle and based on the peak analysis of the signal obtained from luminosity over time. A second-order Butterworth filter and a fast Fourier transformation (FFT) are performed after this signal is obtained. This process provides HR values (BPM) every 0.5 s [26]. The ROI was tracked using a feature tracking algorithm described above (Section 2.2.2). The areas analysed as ROI for HR assessment were the ‘eye area’, ‘forehead’ and ‘face’. This selection was based on the areas that some studies have used as ROI when implementing computer algorithms to analyse HR in humans [17,25].

#### 2.2.4. Data Collected

The protocol of this study was designed to compare invasively and remotely obtained data, collected simultaneously from the same animals during six periods across two consecutive days. The periods recorded included two different conditions (restraint within the crush and during robotic milking).

Physiological parameters included in this study (body temperature, RR and HR) were assessed simultaneously by a gold-standard (labelled as ‘invasive’ for the purpose of this study) and a CV technique. The body temperature of these animals was assessed by the analysis of eye and ear-base temperature obtained from TIR images (frame rate: 1 per second), and by vaginal temperature obtained from temperature loggers (Thermochron^®^; Maxim Integrated Products, Sunnyvale, CA, USA) which were set to record every 60 s, accommodated in a blank controlled internal drug release (CIDR; Pfizer Animal Health, Sterling, CO, USA) device and placed into each participating cow [48]. RR was measured using the respective CV method and by visual observations from RGB video obtained with a Raspberry Pi Camera when cows were in the crush. These visual observations were always performed by the same observer, who counted breathing movements during one minute. Finally, HR of cows was measured using the proposed CV method as well as with a commercial heart rate monitor (Polar WearLink^®^; Polar Electro Oy, Kempele, Finland) as the ‘invasive’ technique, which has been validated and used in cattle in several studies [21,49,50].

One of the built devices (including TIR and RGB cameras) was held outside the crush to record the face of cows from the front (within 1.5 m) and next to them (within 0.5 m). Another of these devices was attached next to the milking robot to record one side of the face of cows (within 0.5 m) during the milking process (Due to the structure of the milking robot and the size of this device it was not possible to place it in front of the face of cows). In addition, two GoPro cameras were attached to the milking robot to record videos from the front and side of the face of cows (within 0.5 m). Consequently, thermal images were obtained from the front of the animals within a distance of 1.5 m (in the crush), and next to the animals’ face within a distance of 0.5 m (in the crush and in the milking machine). While, RGB videos were recorded from three different positions: in front of cows within a distance of 1.5 m, in front of cows within a distance of 0.5 m (in the milking machine), and alongside within a distance of 0.5 m (in the crush and in the milking machine). In the case of non-radiometric IR videos, these were obtained in front of the animals from a distance of 1.5 m (in the crush).

#### 2.2.5. Experiment Procedures

**Figure 3:**
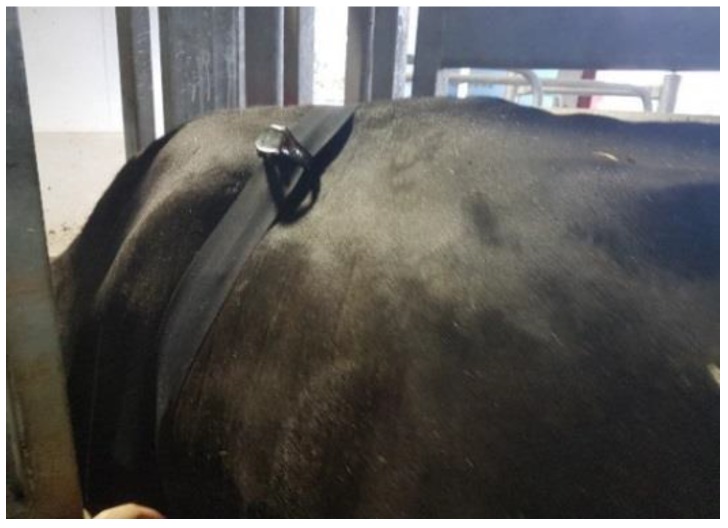
Polar heart rate sensor and Polar watch fastened by elastic band.

**Figure 4:**
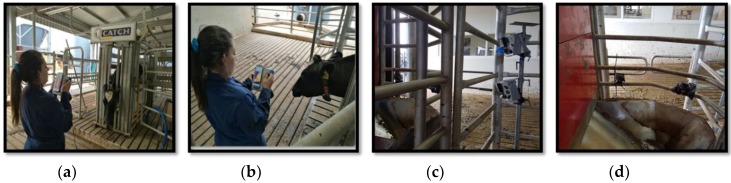
The various camera positions used; (**a**) the developed device is held in front of the animals, within a distance of 1.5 m, (**b**) the developed device is held next to the animals, within a distance of 0.5 m, (**c**) developed devices are located next to the milking robot, within a distance of 0.5 m and (**d**) GoPro cameras are located in the milking robot, within a distance of 0.5 m.

As the first procedure, the group of cows was drafted to a yard that was connected to the crush via a raceway, from where cows were individually moved into the crush. In this position, a patch on the left anterior thorax was shaved, which has been considered as the correct place to measure heart rate in bovines [51,52]. Then, the heart rate monitor was placed using an elastic band around the thorax of the cow, and lubricated with gel (Figure 3). When the HR monitor was attached, the head of the cow was restraint in the head bail for 4 min. The recording was performed in front of the cow’s face the first 3 min, and by the side of the cow’s head during the last minute (Figure 4a,b). After this period, the cow was released from the crush, allowing her to visit the milking robot to be milked.

Cameras were also placed in the milking robot in order to record videos and IR images of the face of these cows when they were being milked (duration 6–11 min) (Figure 4c,d). When the cow finished the milking process, she was moved to the crush again in order to record images similarly to the previous time. Once this recording was finished, the HR monitor was removed and the cow was released on pasture with the rest of the herd during the afternoon. These procedures were performed during the morning (7:00–11:00) in two consecutive days. Non-radiometric IR videos of ten cows were recorded for one minute during the first crush procedure on the first day of the experiment (due to technical difficulties, it was not possible to record during the following procedures). Finally, the CIDR was removed at the end of the last day at the time when the Polar HR monitor was removed.

### 2.3. Statistical Analysis

The accuracy of the feature tracking method was evaluated by measuring the number of frames in which this algorithm correctly tracked the desired area over the total frames processed.

Once the images were processed for the assessment of physiological parameters, statistical analyses were performed using Minitab^®^ Statistical Software 18 (Minitab Pty Ltd., Sydney NSW, Australia). Firstly, normality tests were performed in order to check the distribution of the data obtained. After that, Pearson correlation was calculated to measure the strength of the linear association between each remotely measured parameter (surface temperature, RR and HR) with its respective parameter measured by an ‘invasive’ method (intravaginal temperature loggers, visual observations for RR assessment, and Polar HR monitors). In addition, a linear regression analysis was performed to determine whether remotely measured parameters could accurately predict invasively measured parameters. In terms of temperature and HR, correlations were firstly made with the set of data obtained from the whole group and considering the average of every minute recorded as well as considering the average obtained for each handling procedure (1st crush, milking and 2nd crush). Then, the correlation analyses were performed per cow. From these last correlations, the average and standard deviation (SD) of the correlation coefficients obtained from each setting (type of camera; camera position; camera distance; area of face analysed) were calculated and presented as ‘mean ± SD’.

Furthermore, the differences between the parameters obtained from both methods were calculated. 

## 3. Results

The data obtained from ‘invasive’ and remote methods were compared within the group and within individual animals. 

Firstly, the accuracy of the feature tracking algorithm was calculated. Table 1 displays its level of accuracy when tracking different areas of a cows’ head. This method showed a high accuracy tracking the three areas analysed (92%–95%). Moreover, it improved in accuracy as the size of the area tracked increased.

**Figure 5:**
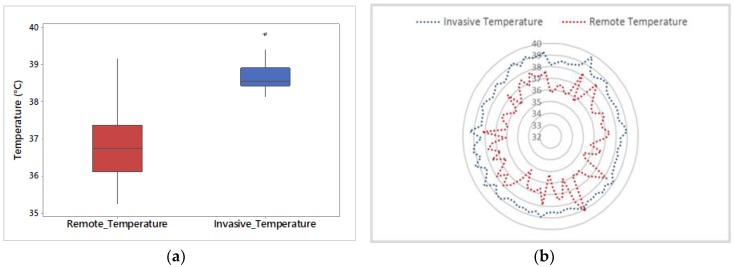
Comparison between intravaginal temperature (Invasive Temperature) and eye temperature obtained from TIR images (Remote Temperature) using (**a**) boxplots and (**b**) a trend graph. For the measurement of remote temperature, the thermal infrared camera (FLIR^®^ AX8) was located beside the animals within a distance of 0.5 m. Data used was the average for each cow within a handling period (milking or crush). N = 60. On a boxplot (a), asterisks (*) denote outliers.

When comparing intravaginal temperatures with eye temperatures, eye temperature had a larger variability than the vaginal temperature (Figure 5a). Furthermore, Figure 5b shows the trend of intravaginal and eye temperature, which shows that eye temperature was permanently lower than intravaginal temperature.

**Figure 6:**
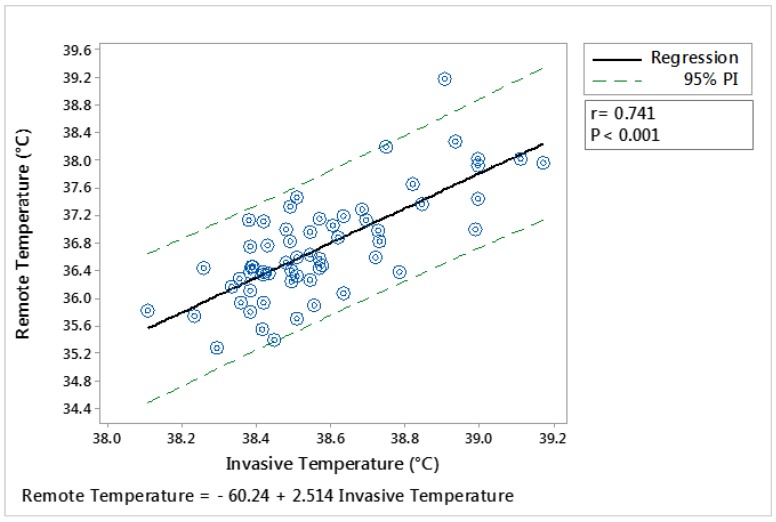
Regression analysis of the relationship between (i) intravaginal temperature (Invasive Temperature) and (ii) eye temperature obtained from thermal infrared images (Remote Temperature). For the measurement of remote temperature, the thermal infrared camera (FLIR^®^ AX8) was located beside the animals within a distance of 0.5 m. The solid line shows the line of best fit, the dotted lines show the 95% and the equation and associated r and *p* value are shown. Each point represents an average for the animal within a handling period (milking or crush). N = 60.

When the analysis among temperatures was performed including all temperature data obtained from the whole group, eye temperature extracted from images that were recorded on the side of cows within 0.5 m of distance, showed a high correlation with intravaginal temperature (r = 0.74; *p* < 0.001; Figure 6). 

The temperature data were then analysed per cow, where correlations were higher for the analysis that used the average of temperature obtained during each handling period (Table 2, Table 3), compared to when the analysis was performed considering the average temperature obtained for each minute recorded. In addition, the highest correlations between invasively and remotely obtained temperatures were observed when ‘eye area’ was selected as the ROI during the processing of TIR images (r = 0.64–0.93), compared to the correlation obtained when ‘ear-base’ was used as ROI (r = 0.33–0.70). 

Individual correlations between eye temperature and intravaginal temperature were higher when images were recorded on the side of the animal within a distance of 0.5 m (r = 0.8 ± 0.06 (mean ± SD); *p* < 0.01), than when images were obtained from the front. On the other hand, remote temperatures obtained from the ear base showed a moderate correlation with intravaginal temperature (r = 0.54 ± 0.15 (mean ± SD); *p* > 0.05) when comparing the temperature averages obtained during each handling period.

The absolute differences between ‘invasive’ and remote temperatures were analysed per individual animal. Eye temperature from front images was identified to be on average 2.4 ± 0.8 °C (mean ± SD) lower than the intravaginal temperature, while eye temperature extracted from images recorded on the side of animals was on average 1.1 ± 0.8 °C (mean ± SD) lower than intravaginal temperature. In addition, ear-base temperature was on average 3.1 ± 0.7 °C (mean ± SD) lower than intravaginal temperature.

**Figure 7:**
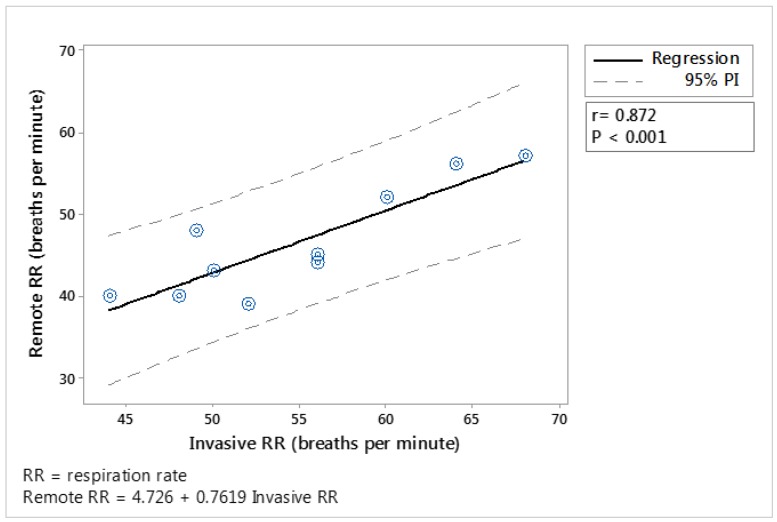
Regression analysis of the relationship between respiration rate (RR) obtained from visual observations (Invasive RR) and the respiration rate remotely obtained (remote RR). For the measurement of remote RR, the camera (FLIR^®^ ONE) was located in front of the animal within a distance of 1.5 m, while the animal was in the first crush. The solid line shows the line of best fit, the dotted lines show the 95% and the equation and associated r and *p*-value are shown. Each point represents an average for the animal while in the crush. N = 10.

In the case of RR assessment, the ten observations obtained from each method (‘invasive’ and remote method) during the first time cows were restrained in the crush were compared through linear regression, and showed a high correlation (r = 0.87; *p* < 0.001; Figure 7). The RR parameters obtained from CV techniques were on average 8.4 ± 3.4 (mean ± SD) breaths per minute lower than the RR parameters obtained by visual observations.

Following this analysis, the HR data were evaluated. Due to inconsistences observed in some of the Polar monitors, the HR data obtained from two animals were not used in this study. The exclusion of this data was based on the large standard deviation (SD) observed. Hopster and Blokhuis [53], and Janzekovic et al. [50] reported a SD of 1.44–6.39 bpm and 6.27–13.92 bpm from the HR of cows obtained from ‘Polar^®^ Sport Testers; therefore, it was decided that data would be excluded from the analysis when it displayed a SD over 20 bpm.

**Figure 8:**
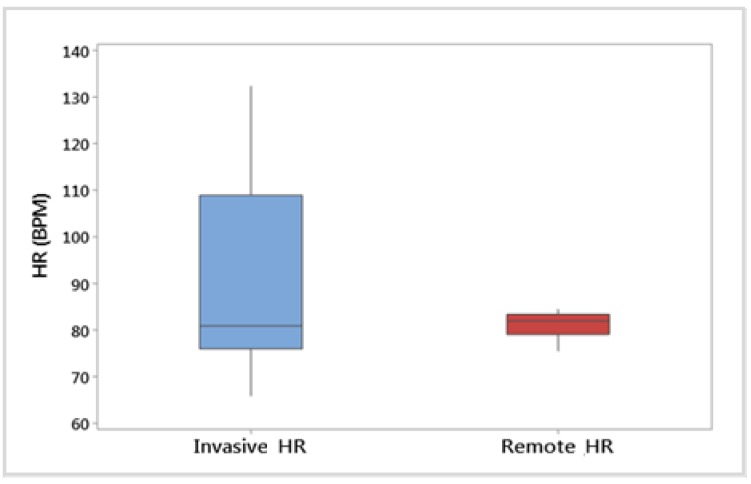
Data distribution for heart rate (HR, beats per minute, BPM) obtained from Polar HR monitors (Invasive HR) and from videos recorded with a Raspberry-pi camera, with the camera in the front within a distance of 1.5 m, while the animal was in the crush, and analysing the ‘eye area’ (Remote HR). Each data point was an average for the animal across all crush handling periods. N = 32.

As Figure 8 shows, the HR obtained invasively had a higher variability than the remote measurements. However, similar medians were obtained from both methods (80.9 and 81.9, respectively). 

**Figure 9:**
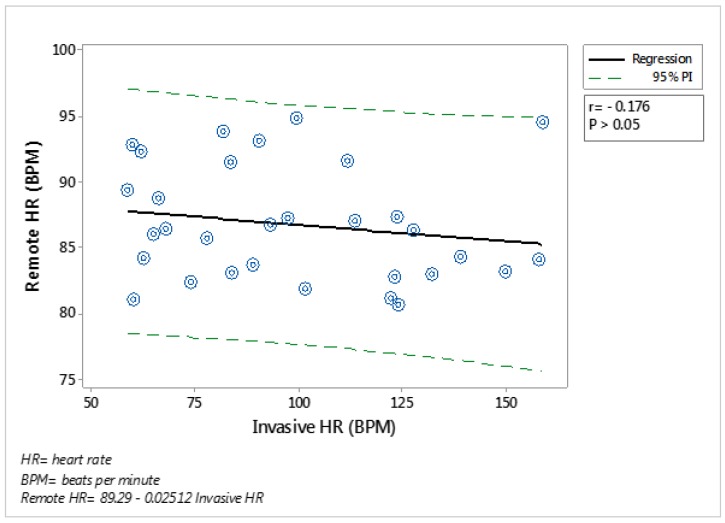
Regression analysis of the relationship between heart rate (HR) obtained from Polar HR monitors and from videos recorded with a Raspberry-pi camera located in front of the animal, within a distance of 1.5 m, while the animal was in the crush, and analysing the ‘eye area’. The solid line shows the line of best fit, the dotted lines show the 95% and the equation and associated r and *p*-value are shown. Each point represents an average for the group within the each crush handling period. N = 32.

No correlations (*p* > 0.05) were found between heart rate recorded by the Polar HRM and analysed from the RGB cameras when the analysis was performed including all HR data obtained from the whole group (Figure 9).

The correlation between methods was then compared within the animal, for the average for each handling period (Table 4), and within the animal, using the average heart rate for each minute across all handling periods (Table 5). The highest correlation was found when comparing the HR average obtained using both methods during each handling procedure (Table 4), in comparison to the analysis that compared the HR average obtained per minute recorded (Table 5). In addition, the highest positive correlations between HR obtained invasively and remotely were observed when ‘eye area’ was selected as the ROI during the processing of RGB videos obtained with Raspberry Pi and GoPro cameras (r = 0.75–0.89; Table 4, Table 5). The mean of the individual correlations was higher when RGB videos were recorded with Raspberry Pi Cameras located in front of the animal within a distance of 1.5 m (r = 0.89 ± 0.09 (mean ± SD); *p* < 0.05; Table 4). On the other hand, the lowest mean of individual correlations, which was not significant, was observed when the ‘face’ was selected as the ROI (r = 0.16 ± 0.2 (mean ± SD); *p* > 0.05; Table 4).

## 4. Discussion

Although changes of physiological parameters have largely been used to detect stress and illness in animals, their assessment still includes some invasive methods that can elevate stress and pain in animals, affecting results and probably animal wellbeing, as well as being time-consuming and labour-intensive. Computer vision techniques could assist animal monitoring and provide valuable information for animal wellbeing assessment. Developed computer algorithms were evaluated to track specific features of cows on the RGB videos, measure surface temperature, RR and HR in cattle.

Animal recognition and tracking is an area of investigation that has become relevant due its contribution to the development of automatic animal monitoring systems [54,55]. This study developed and implemented a feature tracking algorithm in order to improve image processing. This algorithm showed great accuracy when tracking three different areas of the face of the cow (92%–95%). This algorithm had a similar accuracy to the method that Taheri and Toygar [56] proposed to detect and classify animal faces from images (95.31%). However, their method was not applied in moving images. Furthermore, the developed algorithm showed a higher accuracy than the accuracy reported by Jaddoa et al. [57], who tracked the eyes of cattle from TIR images (68%–90% of effectiveness) and was similar to the average accuracy shown by Magee [58], who classified cows from videos by the mark in their body (97% accuracy average).

In terms of the temperatures obtained invasively and remotely during this study, they were compared in order to evaluate the performance of thermal imagery to detect temperature changes in cattle. Following what was observed from the HR analysis of the current study and the observations reported by Hoffmann et al. [6], the comparison between methods was carried out with the data obtained from the whole group, as well as with each cow’s individual temperatures. The current study shows a better performance from the measurements of eye temperature than from ear-base temperature, which is supported by several studies that have used eye temperature of animals with positive outcomes [11,59,60]. The correlation coefficients resulted in this study when comparing vaginal and eye temperatures (r = 0.64–0.80) are higher than the correlation coefficient showed by George et al. [11] (r = 0.52) and Martello et al. [61] (r = 0.43) when compared with vaginal and rectal temperature of cattle, respectively. However, ear-base temperature may be a useful measure when the images are obtained from above, and the eye area is not visible [62]. This has been observed in some studies, where ear-base appeared to be a useful measure when assessing changes of temperature in pigs [19,47].

The correlation between ‘invasive’ and remote temperature measurement was higher when the analysis was run per individual. This can be related to the individual effects, including the individual changes and the level of reactivity to stimuli of each animal, mentioned by Marchant-Forde et al. [63] and Hoffmann et al. [6]. In addition, it is hypothesized that the higher correlation observed when the analysis was performed with the mean temperature obtained within a handling period is related to a lower sensitivity to temperature changes from the vaginal loggers compared to the sensitivity from thermal imagery. Related to this, George et al. [11] argued that the relationship between temperatures obtained from vaginal loggers and TIR images could be affected by the sampling frequency and sensitivity of the techniques. In the case of the current study, the difference of sampling frequency between the temperature loggers (one recording every minute) and the thermal cameras (one image every second) could have had an impact on the correlations obtained. In addition, Stewart et al. [64] claimed that TIR imagery is very sensitive, detecting photons that are emitted in substantial amounts even when there are small changes of temperature in animals, explaining why remotely obtained temperatures seemed to be more sensitive to changes than the temperatures obtained by intravaginal loggers. Furthermore, some researchers have observed that in contrast to intravaginal or rectal temperature, eye temperature appears to decrease immediately after a stressful situation, which is followed later by an increase [59]. Although this decrease was not observed in this study, it could have occurred when animals were being moved to the holding yard just before the crush procedure. This initial decrease and other variabilities of surface temperature can also be due to the natural thermoregulatory mechanism of animals, which can modify the flow of blood through the skin as an adaptative response to changes in ambient temperature, illness and stress among others [65,66]. 

Furthermore, the results from this study show that a shorter distance between the animal and the thermal cameras results in a more accurate assessment of eye temperature. Apart from the correlation being higher between vaginal loggers and TIR imagery when thermal cameras are located on the side of the animal within 0.5 m of distance, the difference between these measurements is lower than when these images are recorded within 1.5 m of distance (1.1 ± 0.8 and 2.4 ± 0.8, respectively). Differences of performance dependent on distance from subject and camera location have been also observed by Church et al. [60] and Jiao et al. [67], who pointed out the importance of a correct and constant camera location to obtain consistent and accurate measurements from TIR images. Similarly to the current results, Church et al. [60] observed a better performance of TIR imagery when the eye-camera distance was up to 1 m. 

Due to the effect that the external conditions and the physiological status of animals have on the temperatures obtained from TIR images, it is important to consider that variations can be observed in the relationship between core body temperature and surface temperature. 

As RR is another relevant parameter for animal monitoring, research is being carried out in order to develop accurate and practical methods to assess it. This study showed positive results that could open the opportunity for further research and implementation of TIR imagery as a remote technique to assess RR of animals. Researchers, such as Stewart et al. [31] have also investigated the potential of TIR imagery to measure RR in cattle. Although they used radiometric IR videos to observe the changes around cows’ nostrils and manually counted their RR, while this study implemented non-radiometric IR videos and a developed algorithm to semi-automatically count cows’ RR, both studies observed good agreement between the proposed methods and visual observation. In addition, both studies showed similar differences between the RR means obtained from compared methods (2.4–8.4 and 8.4 ± 3.4 breaths per minute, respectively). However, Stewart et al. [31] reported that respiration rates from IR images were overestimated when compared to the RR obtained from visual observations, while the present study showed that the respiration rates from IR images were underestimated. These results suggest a promising opportunity to continue the research focused on the improvement of CV techniques based on TIR to automatically assess RR in animals.

The evaluation of HR parameters showed a larger variability in the measurements obtained from the Polar monitors (range: 66–132 bpm), in comparison with the variability observed from the HR remotely obtained (range: 75–102 bpm). These variabilities were similar to what has been observed in other studies. For instance, Janzekovic et al. [50] observed a range of 60–115 bpm from Polar monitors that measured HR of cows during several milking periods. In addition, Wenzel et al. [49] observed a range of 70–103 bpm when measuring HR in dairy cows before and during being milked. However, this difference of variability between remotely and invasively obtained HR differs from the results obtained by Balakrishnan et al. [68], who observed a similar distribution of the HR of humans, when comparing the measurements obtained from ECG and RGB videos. The difference of variability between methods and the narrow range of HR obtained from the proposed CV technique, could indicate that further research is needed to adjust this method to the specific characteristics of cattle.

As Janzekovic et al. [50] suggested, the changes of HR parameters observed in this study appeared to be greatly influenced by the individual response of the animal. This could indicate that measuring HR changes of individuals could be a more precise assessment than only measuring HR per se. Furthermore, as Hopster and Blokhuis [53] identified, the performance of Polar monitors appeared to differ. Taking this into consideration, the comparison between methods was carried out per animal. From this analysis, the highest correlation coefficient resulted from the comparison between the ‘invasive’ and remote HR measurements (r = 0.89 ± 0.09; mean ± SD) and was slightly lower than the correlation observed by Takano and Ohta [9], who reported a correlation coefficient of 0.90 when comparing the human HR provided by pulse oximeters and the HR extracted by CV techniques that identified the change of brightness within the ROI (cheek). However, it was higher than the correlation reported by Cheng et al. [69] when evaluating computer algorithms to assess human HR from RGB videos (r = 0.53).

Some limitations were encountered during the evaluation of HR, which lead to recommendations for improvements in further research. Firstly, similarly to what some authors have reported [25,69], the light conditions and excessive animal motion could have had an impact on the current results. Moreover, as the proposed method involves the detection of brightness changes within the pixels of the ROI, it is suspected that the variation in colour in faces of cows could lead to artefacts in the HR assessment. Considering this, further research is suggested to adjust this method to the different colours and combination of colours present in cows. Secondly, Polar monitors have shown some inconsistency in their performance when used on cattle, which led to some noise in their HR measurements. Similar issues were reported by other researchers, who mentioned that monitors performance was affected by electrodes position and animals movement [53,63]. Marchant-Forde et al. [63] also found a delay in the HR changes observed on the monitors data, compared to the HR changes showed by the ECG, which had a slight effect on the correlation between methods. They also observed that the correlation between methods was greatly dependent on individual animal effects. All these factors could explain why the present study showed better correlations between Polar monitors and computer vision heart rates when the analysis was performed by handling period instead of by minute, and per individual animal instead of the whole group of animals.

A large number of studies in humans and some studies in animals suggested the potential of computer vision techniques to perform several tasks that can contribute to human and animal monitoring. This study shows a promising potential for computer vision techniques to monitor changes in physiological parameters of cattle and other animals, promoting further research focused on improving these techniques to generate useful tools for the assessment of animal health and welfare.

## 5. Conclusions

This study involved the development and validation of computer vision techniques in order to evaluate the potential of remotely sensed data (TIR and RGB imagery) for assessing heart rate, eye and ear-base temperature, and respiration rate in cattle. Although some limitations were identified during the comparison of ‘invasive’ and remote methods, the methods evaluated showed the ability to detect changes in physiological parameters in individual animals. These techniques could lead to the development of useful methods to constantly monitor farm animals and alert any relevant physiological changes on them. Nevertheless, more research is needed to investigate the feasibility of implementing these methods on a larger scale and to decrease the impact that environmental and animal factors have on these measurements.

## Figures and Tables

**Table 1:** Analysis of the accuracy of the tracking method for each feature (eyes, forehead, face).

Tracked Feature	Number of Frames Analysed	Number of Frames Correctly Tracked	Accuracy (%)
Eyes	134,966	124,207	92.0
Forehead	134,966	124,526	92.3
Face	125,715	118,813	94.5

**Table 2:** Pearson correlation coefficients (r) between the core temperature of individual animals obtained from intravaginal loggers and temperature measured using thermal infrared images, analyzing different areas on the animal (eye area, ear base) and different camera positions relative to the animal (front, side, 1.5 m, 0.5 m). Data used is the average for the animal within a period of handling (six handling periods per animal).

Invasive Method	Computer-Vision Method				Mean Correlation Coefficient (r) *	Range (r)	*p*-Value **
	Camera	Position	Distance	Analysed Area			
Thermochron	FLIR AX8	Front	1.5 m	Eye area	0.77 ± 0.12	0.64–0.93	<0.01
Thermochron	FLIR AX8	Side	0.5 m	Eye area	0.8 ± 0.06	0.74–0.89	<0.01
Thermochron	FLIR AX8	Side	0.5 m	Ear base	0.54 ± 0.15	0.33–0.70	<0.05

* Correlation coefficients are presented as mean ± SD; ** *p*-value are the highest observed in each category.

**Table 3:** Pearson correlation coefficients (r) within individual animals, between the core temperature obtained from intravaginal loggers and, temperature measured using thermal infrared images, analyzing different areas on the animal (eye area, ear base) and different camera positions relative to the animal (front, side, 1.5 m, 0.5 m). The data used were the average over one minute, for each animal, within all the handling periods (milking and crush).

Invasive Method	Computer-Vision Method				Mean Correlation Coefficient (r)*	Range (r)	*p*-Value**
	Camera	Position	Distance	Analysed Area			
Thermochron	FLIR AX8	Front	1.5 m	Eye area	0.64 ± 0.18	0.42–0.83	<0.05
Thermochron	FLIR AX8	Side	0.5 m	Eye area	0.68 ± 0.17	0.47–0.86	<0.05
Thermochron	FLIR AX8	Side	0.5 m	Ear base	0.43 ± 0.23	0.33–0.67	<0.05

* Correlation coefficients are presented as mean ± SD; ** *p*-value are the highest observed in each category.

**Table 4:** Pearson correlation coefficients (r) between heart rate measurements of individual animals obtained from Polar monitors and, heart rate measured using computer vision techniques using different cameras (GoPro, Raspberry Pi), different areas on the animal (eye area, forehead, face) and with cameras located within different distances from the animal (1.5 m, 0.5 m). Data are the average for the animal within each period of handling (six handling periods per animal).

Invasive Method	Computer-Vision Method				Mean Correlation Coefficient (r) *	Range (r)	*p*-Value **
	Camera	Position	Distance	Analysed Area			
Polar monitor	RaspberryPi	Front	1.5 m	Eye area	0.89 ± 0.09	0.72–0.99	<0.05
Polar monitor	RaspberryPi	Front	1.5 m	Forehead	0.62 ± 0.23	0.32–0.90	<0.05
Polar monitor	RaspberryPi	Front	1.5 m	Face	0.16 ± 0.2	−0.11–0.39	>0.05
Polar monitor	RaspberryPi	Side	0.5 m	Eye area	0.78 ± 0.04	0.74–0.84	<0.01
Polar monitor	RaspberryPi	Side	0.5 m	Forehead	0.71 ± 0.18	0.53–0.89	<0.05
Polar monitor	GoPro	Side	0.5 m	Eye area	0.75 ± 0.14	0.55–0.92	<0.05
Polar monitor	GoPro	Front	0.5 m	Forehead	0.65 ± 0.08	0.58–0.76	<0.05

* Correlation coefficients are presented as mean ± SD; ** *p*-value are the highest observed in each category.

**Table 5:** Pearson correlation coefficients (r) between heart rate measurements of individual animals, obtained from Polar monitors and, heart rate measured using computer vision techniques using different cameras (GoPro, Raspberry Pi), different areas on the animal (eye area, forehead, face) and with cameras located within different distances from the animal (1.5 m, 0.5 m). The data used were the average over one minute, within all the handling periods (milking and crush).

Invasive method	Computer-Vision Method				Mean Correlation Coefficient (r) *	Range (r)	*p*-Value **
	Camera	Position	Distance	Analysed Area			
Polar monitor	RaspberryPi	Front	1.5 m	Eye area	0.83 ± 0.15	0.55–0.99	<0.05
Polar monitor	RaspberryPi	Front	1.5 m	Forehead	0.78 ± 0.19	0.41–0.99	<0.05
Polar monitor	RaspberryPi	Front	1.5 m	Face	0.2 ± 0.23	−0.1–0.4	>0.05
Polar monitor	RaspberryPi	Side	0.5 m	Eye area	0.75 ± 0.19	0.46–0.96	<0.01
Polar monitor	RaspberryPi	Side	0.5 m	Forehead	0.77 ± 0.20	0.39–0.98	<0.05
Polar monitor	GoPro	Side	0.5 m	Eye area	0.77 ± 0.11	0.61–0.87	<0.01
Polar monitor	GoPro	Front	0.5 m	Forehead	0.79 ± 0.18	0.50–0.99	<0.05

* Correlation coefficients are presented as mean ± SD; ** *p*-value are the highest observed in each category.
